# Obstetric and perinatal outcomes in parturients with active SARS-CoV-2 infection during labor and delivery: a retrospective cohort study

**DOI:** 10.1186/s12884-022-04825-6

**Published:** 2022-06-23

**Authors:** Rita Zlatkin, Sarah Dollinger, Chen Jacoby, Anat Shmueli, Shiri Barbash-Hazan, Rony Chen, Arnon Wiznitzer, Eran Hadar

**Affiliations:** 1 Helen Schneider Hospital for Women, Rabin Medical Center – Beilinson Hospital, 39 Jabotinsky St., Petach Tikva, Israel; 2 Sackler Faculty of Medicine, Tel Aviv University, 4941492 Tel Aviv, Israel

**Keywords:** SARS-CoV-2, COVID-19, Pregnancy, Delivery, Neonatal outcomes

## Abstract

**Background:**

The COVID-19 pandemic is an ongoing global healthcare crisis that negatively affects pregnant women. Although patients with an acute infection during pregnancy have been widely studied, information regarding labor and delivery while infected is sparse. The aim of the study was to ascertain maternal, obstetrical, and perinatal outcomes of women who gave birth while infected with SARS-CoV-2.

**Methods:**

Patients diagnosed with COVID-19 during pregnancy at a tertiary medical center in 4/20–2/21 were identified by a retrospective database search. Those with an active intrapartum SARS-CoV-2 infection were compared with those who recovered at least 10 days before labor and delivery.

**Results:**

Of the 176 women included in the study, 84 had a SARS-CoV-2 infection at the time of delivery and 92 had recovered from the infection. There was no statistically significant between-group difference in mean gestational age at delivery (39 weeks for both, *p* = 0.71) and overall rate of cesarean delivery (26.2% vs 17.4%, respectively, *p* = 0.35) or non-elective cesarean delivery (10.71% vs 4.34%, respectively, *p* = 0.48). In the active-infection group, the rate of severe disease was 2.4%, and of critical disease (with intensive care unit admission, mechanical ventilation, and ECMO), 3.6%, compared to zero for both in the recovered group. No differences were found between the groups in adverse perinatal outcomes.

**Conclusion:**

Delivery is safe and feasible in women with active SARS-CoV-2 infection. Nevertheless, we found a non-significant trend for more severe disease and for cesarean delivery and urgent cesarean delivery (for COVID-19-related indications) in women with an intrapartum SARS-CoV-2 infection.

## Introduction

The coronavirus disease 2019 (COVID-19) is caused by a single-stranded RNA virus, severe acute respiratory syndrome coronavirus 2 (SARS-CoV-2) [1]. The World Health Organization declared the COVID-19 outbreak a pandemic in March 2020. It was assumed early on that, like for other infections due to respiratory viruses and related coronavirus strains, pregnant women were more vulnerable to SARS-CoV-2 infection and at higher risk of severe disease and complications than the general population [2, 3]. Investigations regarding the effect of COVID-19 infection on maternal and fetal outcomes are still ongoing [4]. Of note, there are several reports of higher rates of admission to intensive care units (ICU), preterm birth, cesarean delivery, and preeclampsia in SARS-CoV-2-infected women [5–10]. Furthermore, despite the many studies on pregnancy and SARS-CoV-2 infection, the literature on parturients with an active infection during delivery is sparse. Data are lacking on the potential impact of the acute infection, or the isolated environmental setting, on different aspects of the delivery process, mainly the mode of delivery, and possibly other outcome factors.

The aim of the present study was to examine maternal, obstetrical, and perinatal outcomes of women with an active SARS-CoV-2 infection at the time of delivery compared to patients who recovered from COVID-19 during pregnancy. The composition of the control group was based on a previous study by Fan et al. [11] which found no adverse or neonatal effects in COVID-19-recovered parturients.

## Methods

### Study population

All pregnant women who gave birth at a tertiary medical center between April 2020 and February 2021 and were infected with COVID-19 during pregnancy were identified by retrospective search of the hospital’s electronic database. The cohort was divided into two groups: those who tested positive for SARS-CoV-2 up to 10 days prior to delivery and with those who had recovered from the infection by the time of delivery.

### Setting

The intrapartum setting for women who are acutely infected with SARS-CoV-2 is significantly different from that of other parturients. They give birth in a positively pressured delivery room without escorts and are obligated to wear a disposable non-woven face mask. Each patient is supervised by a dedicated midwife who is present in the COVID-designated delivery room or observed via a sound and video monitor by the delivery room medical staff. All caregivers who are in close contact with the patient are obligated to use personal protective equipment, including gloves, isolation gown, N95 filtering face-piece, respirator, and face shield.

### Definitions

The diagnosis of SARS-CoV-2 infection (Alpha and Delta variants) was based on a positive result of real-time reverse transcriptase polymerase-chain-reaction (RT-PCR) assay of a nasopharyngeal swab specimen (Seegene, Songpa-gu, South Korea). Pregnant women in our center were routinely screened for SARS-CoV-2 if they were in active labor, admitted to the hospital for any reason, quarantined due to exposure to a known COVID-19 patient, and/or had signs or symptoms related to COVID-19. All neonates born to SARS-CoV-2-positive parturients were tested for the virus at 24 and 48 h after birth. Our institution’s policy was to recommend for seperation between the mother and neonate after birth in order to prevent neonatal infection, however it was not mandatory.

Recovery from COVID-19 was defined according to the criteria of the Israel Ministry of Health [12]: at least 10 days had passed since the initial positive test (or borderline-positive test with a confimatory PCR result) and the patient had had none of the following symptoms for at least 3 days: fever ≥ 38 °C, dyspnea or cough or any other respiratory symptom that did not subside, vomiting, and diarrhea.

The severity of COVID-19 disease was ascertained according to the criteria of Wu et al. [13], as follows: mild – any symptoms related to COVID-19; severe – tachypnea (respiratory rate > 30), peripheral oxygen saturation less than 94%, and/or significant lung inflitratres; critical – respiratory failure, septic shock, or mutiple organ dysfunction.

Grand multiparity was defined as parity equal to or greater than five. Preterm birth was defined as delivery before 37 gestational weeks. Birthweight percentiles were calculated using a nationally accepted, gender-specific, reference growth curve [14]. Large for gestational age (LGA) was defined as birthweight above the 90^th^ percentile for gestational age, and small for gestational age (SGA), defined as birthweight below the 10^th^ percentile for gestatonal age.

For the present study, we documented the lowest peripheral oxygen saturation level measured during hospitalization and blood tests were performed throughout the peripartum. We present the highest or lowest laboratory values according to clinical relevance. The standard cutoffs of our institution were used for all hematological and biochemical parameters.

Antepartum and/or postpartum treatment for COVID-19 consisted of a prophylactic dose of low-molecular-weight heparin (LMWH), alone for hospitalized symptomatic patients and combined with dexamethasone 6 mg daily for 10 days or until discharge for patients with severe disease.

### Data collection

Data were retrieved from the hospital’s comprehensive computerized maternal and neonatal medical records, including records from the emergency room triage, delivery room, maternal–fetal hospitalization, and neonatal nursery or NICU. The collected data included maternal demographics, medical and obstetrical background, antepartum pregnancy follow-up, SARS-CoV-2 test results, COVID-19 clinical parameters and complications, hematological and biochemical test results in the peripartum period, peripartum and postpartum treatment for COVID-19, and obstetrical and perinatal outcomes.

### Outcome measures

The primary outcome measure of the study was mode of delivery. Secondary outcomes were maternal SARS-CoV-2 infection characteristics (disease severity, ICU admission and mechanical ventilation), preterm birth, perinatal complications (low Apgar score, NICU admission, and neonatal acidemia defined as umbilical cord pH < 7.2) and neonatal SARS-CoV-2 infection.

### Statistical analysis

Data analysis was performed with Statistical Analysis Software version 9.4 (SAS, Cary, NC, USA). Data are presented as median and interquartile range (IQR). Continuous variables were compared using the general linear model. Chi-square and Fisher’s exact tests were used for categorical variables, as appropriate. Differences were considered significant when *p*-value was < 0.05.

### Ethics

The study was approved by the Institutional Review Board of Rabin Medical Center (approval no. 331–20-RMC). Informed consent was waived due to the study’s retrospective design by the Institutional Review Board of Rabin Medical Center. I confirm that all methods were carried out in accordance with all relevant guidelines and regulations.

## Results

A total 176 women met the inclusion criteria: 84 were diagnosed with active intrapartum SARS-CoV-2 infection and 92 had recovered from COVID-19 by the time of labor and delivery. There were no significant between-group differences in baseline demographic and clinical characteristics, as shown in Table 1.

**Table 1:** Clinical and pregnancy characteristics of pregnant women with COVID-19 with or without active disease at delivery

Clinical and pregnancy characteristics	COVID-19- recovered patients	COVID-19-active patients	*p*-value
	***N* = 92**	***N* = 84**	
**Maternal age (years)**	30 (17–45)	30 (20–44)	0.965
**Body mass index (kg/m**^**2**^)*	24.4 (17.9–40.1)	23.5 (17.3–41)	0.754
**Gravidity**	3 (1–14)	3 (1–12)	0.761
**Parity**	1.5 (0–10)	2 (0–9)	0.913
**Nulliparity**	26 (28.26%)	22 (26.19%)	0.943
**Multiparity**	10 (10.87%)	10 (11.9%)	0.943
**Previous cesarean delivery**	14 (15.22%)	14 (16.67%)	0.839
**Coexisting medical diseases**			
** Asthma**	2 (2.15%)	1 (1.2%)	1
** Inflammatory bowel disease**	3 (3.26%)	0 (0%)	0.247
** Diabetes mellitus**	3 (3.26%)	2 (2.38%)	1
** Thyroid disease**	8 (8.7%)	2 (2.38%)	0.103
** Chronic hypertension**	0 (0%)	1 (1.19%)	0.477
** Anxiety disorder**	3 (3.26%)	2 (2.38%)	1
** Antiphospholipid antibodies syndrome**	2 (2.17%)	0 (0%)	0.498
** Chronic medication use**	16 (17.39)	8 (9.52)	0.186
**Mode of conception**			
** Spontaneous**	87 (94.57%)	79 (94.05%)	0.724
** Assisted reproduction**	5 (5.43%)	3 (3.57%)	0.724
** Gestational diabetes mellitus**	13 (14.13%)	15 (17.86%)	0.541
** Hypertensive disorders in pregnancy**	2 (2.17%)	2 (2.38%)	0.134

Values are presented as median (range) for continuous variables and as n (%) for categorical variables

The characteristics of the SARS-CoV-2 infection in the two groups are described in Table 2. Women in the active-infection group were diagnosed at a significantly more advanced gestational age than the recovered group (38.5 vs. 29 gestational weeks, respectively. *p* < 0.0001) and were less symptomatic at the time of initial SARS-CoV-2 diagnosis (33.33% vs. 57.74%, *p* < 0.001). The most common symptoms in the active-infection group were cough (19.05%), malaise (13.1%), and fever (9.52%), and in the recovered group, malaise (38.02%), cough (21.12%) and anosmia/ageusia (18.30%). There was no difference in median peripheral oxygen saturation between the groups. Peripheral oxygen saturation was less than 94% in 6% of the active-infection group but in none of the women in the recovered group (*p* = 0.14). The active infection group also had a significantly higher rate of hospital admission for COVID-19 (9.52% vs. 2.17% in the recovered group, *p* = 0.04), and ICU admission (3.6% vs 0, *p* = 0.1). Three patients in the active-infection group required mechanical ventilation followed by extracorporeal membrane oxygenation (ECMO), whereas none of the patients in the recovered group required these measures (3.6% vs 0, *p* = 0.1). The active-infection group was also characterized by a significantly lower rate of mild COVID-19 disease (27.38% vs. 57.74% in the recovered group, *p* = 0.002), and significantly higher rates of severe and critical disease (2.38% vs 0 and 3.60% vs 0, respectively, *p* = 0.1 for both).

**Table 2:** Characteristics of COVID-19 disease in pregnant women with or without active disease at delivery

**COVID-19 characteristics**	**COVID-19**-r**ecovered patients**	**COVID-19**-a**ctive patients**	***p*-value**
	***N* = 92**	***N* = 84**	
**Gestational age at diagnosis (weeks)**	29 (9–39)	38.5 (25–41)	< 0.0001
Symptomatic COVID-19*	41 (57.74%)	28 (33.33%)	< 0.001
**Symptoms**^**a**^			
**Malaise**	27 (38.02%)	11 (13.10%)	< 0.001
**Cough**	15 (21.12%)	16 (19.05%)	0.84
**Anosmia and/or ageusia**	13 (18.30%)	5 (5.95%)	0.02
**Fever**	12 (16.90%)	8 (9.52%)	0.23
**Dyspnea**	12 (16.90%)	4 (4.76%)	0.01
**Sore throat**	5 (7.04%)	2 (2.38%)	0.24
**Gastrointestinal**	8 (11.26%)	2 (2.38%)	0.04
**Oxygen saturation (%)**^**b**^	98 (94–100)	98 (82–100)	0.78
** < 94%**	0 (0%)	5 (6.0%)	0.14
**COVID-19 severity**			
**Mild**	41 (57.74%)	23 (27.38%)	0.002
**Severe**	0 (0%)	2 (2.38%)	0.50
**Critical**	0 (0%)	3 (3.60%)	0.25
**Hospitalization**	2 (2.17%)	8 (9.52%)	0.04
**ICU admission**	0 (0%)	3 (3.60%)	0.10
**Invasive ventilation**	0 (0%)	3 (3.60%)	0.10
**ECMO**	0 (0%)	3 (3.60%)	0.10

Values are presented as median (range) for continuous variables and as n(%) for categorical variables

*Abbreviations*: *ICU* Intensive care unit, *ECMO* Extracorporeal membrane oxygenation

^a^ Data were missing for 21 women in the recovered group

^b^ Data were missing for 68 women in the recovered group and 47 women in the active- infection group

The laboratory test results in the two groups are presented in Table 3. No significant between-group difference was noted in white blood cell count. The active-infection group had higher rates of lymphopenia (21.43% vs. 3.5%, *p* = 0.003) and thrombocytopenia (34.52% vs. 17.85%, *p* = 0.035). International normalized ratio and fibrinogen levels were similar in the two groups, as were levels of liver enzymes (aspartate aminotransferase and alanine aminotransferase), creatinine, lactate dehydrogenase, and C-reactive protein.

**Table 3:** Peripartum blood analysis in pregnant women with or without active disease at delivery

Blood parameters	COVID-19- recovered patients	COVID-19- active patients	*p*-value
	***N* = 92**	***N* = 84**	
**WBC (K/micL),**^**a**^** highest measured**	12.35 (7.33–20.4)	10.2 (5.69–21.7)	0.0064
**WBC ≥ 15 K/micL**	10 (17.85%)	8 (9.52%)	0.02
**Lymphocytes (K/micL),**^**a**^** lowest measured**	1.7 (0.6–3.7)	1.3 (0.3–3.6)	< 0.0001
Lymphocytes < **1 K/micL**	2 (3.5%)	18 (21.43%)	0.003
**Hemoglobin (g/dL), lowest measured**	11.4 (6.8–14.1)	10.9 (6.2–13.6)	0.09
Hemoglobin < **10 g/dL**	15 (26.78%)	23 (27.38%)	1
**Platelets (K/micL),**^**a**^** lowest measured**	199.5 (85–356)	161 (27–352)	< 0.001
Platelets < **150 K/micL**	10 (17.85%)	29 (34.52%)	0.03
**INR, highest measured**^**b**^	0.95 (0.9–0.99)	0.97 (0.86–1.23)	0.08
INR > **1.1**	0 (0%)	6 (7.14%)	0.57
**Fibrinogen (mg/dL), lowest measured**^**c**^	640.5 (193–833)	587 (156–837)	0.062
Fibrinogen < **200 mg/dL**	1 (5.55%)	2 (2.77%)	0.52
**AST (U/L), highest measured**^**d**^	30 (15–143)	26 (15–178)	0.96
AST > **31 mg/dl**	2 (33.33%)	16 (37.20%)	0.85
**ALT (U/L), highest measured**^**e**^	17 (7–375)	18 (6–111)	0.93
ALT > **34 U/L**	2 (25%)	10 (22.72%)	0.88
**LDH (U/L), highest measured**^**f**^	588.5 (447—892)	526 (350—1288)	0.65
LDH > **600 U/L**	3 (50%)	17 40.47%)	0.65
**CRP (mg/dL), highest measured**^**g**^	16.5	9.25 (1.43—21.72)	0.50
**Creatinine (mg/dL), highest measured)**^**h**^	0.58 (0.52–0.85)	0.52 (0.37–0.85)	0.07

Values are presented as median (range) for continuous variables and as n(%) for categorical variables

*Abbreviations*: *WBC* White blood cells, *INR* International normalized ratio, *AST* Aspartate aminotransferase, *ALT* Alanine aminotransferase, *LDH* Lactate dehydrogenase, *CRP* C-reactive protein

^a^ Complete blood count data were available for only 56 women in the recovered group

^b^ Data were available for 9 women in the recovered group and 42 women in the active- infection group

^c^ Data were available for 18 women in the recovered group and 72 women in the active- infection group

^d^ Data were available for 6 women in the recovered group and 43 in the active-infection group

^e^ Data were available for 8 patients in the recovered group and 44 in the active-infection group

^f^ Data were available for 6 women in the recovered group and 42 in the active-infection group

^g^ Data were available for one woman on the recovered group and 12 women in the active- infection group

^h^ Data were available for 7 women in the recovered group and 45 in the active-infection group

Median D-dimer level in the active-infection group was 2530 ng/mL (range 825–50,422); 14.29% of patients had a D-dimer level of ≥ 3300 ng/mL. D-dimer was not tested in women in the recovered group.

Table 4 shows the obstetric and neonatal outcomes. There was no significant between-group difference in mode of delivery. Compared to the recovered group, the active-infection group had comparable rates of cesarean delivery (26.19% vs. 17.39%, *p* = 0.35) and of non-elective cesarean delivery (10.71% vs 4.34%, *p* = 0.48). The indicationss for non-elective cesarean delivery in the active-infection groups were non-reassuring fetal heart rate in 5 patients, dysfunctional labor in 1, and need for cesarean delivery because of severe COVID-19 in 3. Indications in the recovered group were nonreassuring fetal heart rate in 3 patients and dysfinctional labor in 1. The difference in the rate of non-reassuring heart failure between the groups was not statistically significant (*p* = 0.48). In the 5 of the 9 women in the active-infection group who underwent non-elective cesarean delivery (55.55%), the indication was unrelated to the COVID-19 disease status.

**Table 4:** Obstetric and neonatal outcomes in pregnant women with or without active disease at delivery

Outcome parameters	COVID-19 recovered patients	COVID-19 active patients	*p*-value
	***N* = 92**	***N* = 84**	
**Mode of delivery**			
**Normal vaginal**	73 (79.35%)	59 (70.24%)	0.35
**Assisted vaginal**	3 (3.26%)	3 (3.57%)	0.35
**Cesarean**	16 (17.39%)	22 (26.19%)	0.35
**Indication for** c**esarean delivery**			
**Elective**	12 (13.04%)	13 (15.47%)	0.48
**Non-elective**	4 (4.34%)	9 (10.71%)	0.48
**COVID-19-related indications**	0 (0%)	4 (4.76%)	0.12
**Induction of labor**	17 (18.48%)	17 (20.24%)	1
**Gestational age at delivery (weeks)**	39 (30–41)	39 (28–41)	0.71
**Preterm** b**irth (< 37 weeks)**	10 (10.87%)	7 (8.33%)	0.61
**Length of hospitalization (days)**	4 (2–40)	4 (2–60)	0.77
**Antepartum treatment**	2 (2.17%)	3 (3.57%)	0.67
**Postpartum treatment**	10 (10.87%)	64 (76.19%)	< 0.001
**Neonatal gender, male**	46 (50%)	31 (36.9%)	0.09
**Birthweight (grams)**	3162 (1250–4206)	3214 (780–4082)	0.42
**Neonatal weight percentile (%)**	53 (3–99)	56 (4–99)	0.53
**Small for gestational age**	8 (8.70%)	5 (5.95%)	0.57
**Large for gestational age**	11 (11.96%)	10 (11.90%)	1
**1-min Apgar < 7**	1 (1.09%)	5 (5.95%)	0.10
**5-min Apgar < 7**	1 (1.09%)	3 (3.57%)	0.35
**Cord pH**	7.32 (6.98–7.52)	7.3 (7.1–7.39)	0.12
**pH < 7.2**	4 (4.35%)	2 (2.38%)	1
**NICU admission**	7 (7.61%)	8 (9.52%)	1
**SARS-CoV-2-positive neonate**	0 (0%)	6 (7.14%)	< 0.001

Values are presented as median (range) for continuous variables and as n(%) for categorical variables

*Abbreviations*: *NICU* Neonatal intensive care unit

No significant differences were found between the active-infection and recovered groups in gestational age at delivery (39 weeks in both, *p* = 0.71), rate of preterm delivery (10.87% vs 8.33%, respectively, *p* = 0.61), and rate of induction of labor (18.48% vs. 20.24%, respectively, *p* = 1). Postpartum treatment with LMWH alone or with dexamethasone was significantly more common in the active-infection group (76.19% vs. 10.87%, *p* < 0.001), but antepartum treatment rates in the two groups were comparable for both drugs. There was no between-group difference in median length of hospitalization.

Comparison of perinatal outcomes between the groups yielded no significant difference in birthweight, birthweight percentile, rate of SGA infants, rate of 1-min and 5-min Apgar score < 7, median umbilical cord pH, and rate of NICU admission. SARS-CoV-2 test was positive in 6 neonates in the active-infection group and in none of the neonates in the recovered group (7.14% vs. 0%, *p* < 0.001).

## Discussion

In the present study, we compared women with an active intrapartum SARS-CoV-2 infection with women who had contracted SARS-CoV-2 infection during pregnancy but recovered by the time of delivery. There were no statistically significant differences between the groups in adverse maternal, obstetrical, or perinatal outcomes with the exception of higher rates of postpartum treatment and of SARS-CoV-2-positive neonates in the active-infection group. Women in the active infection-group showed a trend of higher rates of severe and critical COVID-19 disease, ICU admission, mechanical ventilation, preterm delivery, and emergent caesarean deliveries mostly related to COVID-19 severity (and not for obstetrical indications).

Although the two groups had similar baseline characteristics, their COVID-19 features differed. The active-infection group was composed mostly of asymptomatic women who were diagnosed on routine screening at hospital admission whereas a large proportion of the recovered group had sought medical care for symptoms. However, by the time of delivery, the recovered group was completely asymptomatic whereas 5 women in the active-infection group had severe disease, including 3 (3.6%) with critical disease requiring ICU admission. Overall, the rate of caesarean delivery was very high [15] in the active-infection group, reaching 26.19%, and nearly half these procedures (40.9%) were performed for non-elective indications. By comparison, 17.39% of the recovered group underwent cesarean delivery, and about one-fourth of the procedures (26.2%) were for non-elective indications. The 3 patients in the active-infection group who required treatment in the ICU accounted for about one-fourth of the patients who had a cesarean delivery – which was performed so they could undergo more aggressive treatment for the disease, including prone positioning and ECMO. Thus, it is clear that the severity of disease dictated the mode of delivery. Similarly, three out of seven (42.85%) preterm births in the active infection group were iatrogenic and only induced in order to allow more treatment options for the maternal severe infection.

The WAPM study group reported an astoundingly high rate of 11.1% for ICU admissions in pregnant women with SARS-CoV-2 infection [5] and a meta-analysis by Allotey et al. [16] found that pregnant women with COVID-19 had twice the likelihood of being admitted to the ICU than COVID-19-positive non-pregnant women. Our finding that none of the women in the recovered group were admitted to the ICU supports the notion that at the time of delivery, this group resembled the general pregnant population. This assumption is supported by the finding that rates of caesarean and preterm delivery were lower in the recovered group (17.39% and 10.87%, respectively) than in patients with COVID-19 reported in the literature (33%-91% and 12%-21%, respectively) [9, 17, 18], and were closer to the values reported in the general population (19.1% and 10.6%, respectively) [19, 20]. The majority of these women had full-term deliveries and were hospitalized for a short term thereafter; those hospitalized longer had obstetric indications unrelated to COVID-19.

 We expected that women with COVID-19, even with mild to moderate disease, might be more likely to undergo induction of labor at term because of concerns about disease aggravation. Nevertheless, we found no between-group difference for this parameter. This might be explained by the large proportion of women in the active-infection group who were asymptomatic on presentation to the obstetric emergency room in active labor and were diagnosed only on routine screening according to hospital policy. Therefore, they did not require induction of labor in any case.

Of note, our results show that almost half the women in the recovered group were symptomatic, while the majority of women in the active infection group were asymptomatic. Bearing in mind that symptomatic disease is described as a possible marker for higher risk of perinatal complications [21], it is interesting that our study suggests otherwise.

Parturients with COVID-19, regardless of the status of the infection or severity of the disease, give birth in an isolated delivery room, usually unescorted by a family member. This experience can be difficult, especially for nulliparous women, and might potentially affect the postpartum period. From the caregiver aspect, the physical distancing from the patient along with the logistics required to enter the delivery room and perform the examination, may plausibly lead to unfavourable obstetric outcomes.

Analysis of neonatal parameters yielded no significant differences in median birthweight, birthweight percentile, and proportion of SGA neonates. Rates of SGA were low: 5.95% in the active-infection group and 8.75 in the recovered group. Accordingly, Mullins et al. [22] showed that SGA rates in pregnancies complicated by COVID-19 were comparable to those in in pre-COVID-19 registries.

The 7.4% rate of SARS-CoV-2-positive neonates in the active infection group was higher than the reported 2.5% overall risk of neonatal infection in women with symptomatic disease [23]. This findings can be explained by our screening routine which identifies asymptomatic patients, who are as infectious as their symptomatic counterparts [24]. Additionally, several studies suggested that neonatal infection rates may be higher in women with symptomatic COVID-19 [25–27], possibly because of the higher viral load and longer virus-shedding period which could contribute to viral transmission from mother to newborn [28]. It should also be noted that in our cohort, the majority of neonates born to recovered mothers were not tested for SARS-CoV-2, and those that were tested were frequently only swabbed once. By contrast, all neonates born to actively infected mothers were tested twice, 24 and 48 h after delivery. Therefore, some SARS-CoV-2-positive neonates in the recovered group may have been missed, especially those neonates born to women with a recent infection.

### Strengths and limitations

The main strengths of this study are the methodology and setting. To the best of our knowledge, there are no prior studies comparing women with active COVID-19 at the time of delivery with women who had recovered from the infection. Furthermore, as all pregnant women who are hospitalized at our institution are routinely screened for SARS-CoV-2, the active infection group is representative of the spectrum of disease severity in this population. The recovered group, on the other hand, consisted of women who were infected at any time during pregnancy, and was therefore relatively heterogenic group.

The main limitation of this study is its retrospective design. It was especially challenging to collect data regarding COVID-19 symptoms in the recovered group owing to the risk of recall bias. Additionally, it is possible that our study was underpowered by the small patient groups. Further larger scale studies are needed to corroborate our findings.

## Conclusion

We did not find a statistically significant difference between pregnant patients with an active SARS-CoV-2 infection at delivery and recovered COVID-19 pregnant women in terms of obstetric and perinatal complications. These findings suggest that labor and delivery is safe in women with an active SARS-CoV-2 infection. However, the women with an active infection showed a trend to more severe and critical COVID-19 disease, higher rates of ICU admission and mechanical ventilation, and a higher rate of cesarean delivery, especially caesarean delivery for non-elective, COVID-related indications.

## Data Availability

The datasets used and/or analyzed during the current study are available from the corresponding author on reasonable request.
